# Integrative Network Pharmacology, Molecular Docking, and Dynamics Simulations Reveal the Mechanisms of *Cinnamomum tamala* in Diabetic Nephropathy Treatment: An In Silico Study

**DOI:** 10.3390/cimb46110705

**Published:** 2024-10-23

**Authors:** Rashmi Singh, Nilanchala Sahu, Rama Tyagi, Perwez Alam, Ali Akhtar, Ramanpreet Walia, Amrish Chandra, Swati Madan

**Affiliations:** 1 Amity Institute of Pharmacy, Amity University, Noida 201303, Uttar Pradesh, India; srashmi8126@gmail.com (R.S.); rwalia@amity.edu (R.W.); 2 Metro College of Health Sciences & Research, Greater Noida 201310, Uttar Pradesh, India; 3 Sharda School of Pharmacy, Sharda University, Greater Noida 201310, Uttar Pradesh, India; nilanchalasahu24@gmail.com (N.S.); amrish.chandra@sharda.ac.in (A.C.); 4 Galgotias College of Pharmacy, Greater Noida 201310, Uttar Pradesh, India; tyagirama8@gmail.com; 5 Department of Pharmacognosy, College of Pharmacy, King Saud University, P.O. Box 2457, Riyadh 11451, Saudi Arabia; aakhtar@ksu.edu.sa

**Keywords:** diabetic nephropathy, *Cinnamomum tamala*, IMPPAT database, STRING, molecular docking, molecular dynamics simulation, principal component analysis, network pharmacology, PI3K-AKT signaling pathway, PPAR signaling pathway

## Abstract

Diabetic nephropathy (DN) is a serious diabetes-related complication leading to kidney damage. *Cinnamomum tamala* (CT), traditionally used in managing diabetes and kidney disorders, has shown potential in treating DN, although its active compounds and mechanisms are not fully understood. This study aims to identify CT’s bioactive compounds and explore their therapeutic mechanisms in DN. Active compounds in CT were identified using the Indian Medicinal Plants, Phytochemicals and Therapeutics database, and their potential targets were predicted with PharmMapper. DN-related targets were sourced from GeneCards, and therapeutic targets were identified by intersecting the compound–target and disease–target data. Bioinformatics analyses, including the Kyoto Encyclopedia of Genes and Genomes and Gene Ontology enrichment studies, were performed on these targets. A protein–protein interaction network was constructed using STRING and Cytoscape. Molecular docking and dynamics simulations validated the most promising compound–target interactions. Six active compounds in CT were identified, along with 347 potential therapeutic targets, of which 70 were DN-relevant. Key targets like MMP9, EGFR, and AKT1 were highlighted, and the PPAR and PI3K-AKT signaling pathways were identified as the primary mechanisms through which CT may treat DN. CT shows promise in treating DN by modulating key pathways related to cellular development, inflammation, and metabolism.

## 1. Introduction

Diabetic nephropathy (DN) is an increasingly serious public health issue with significant economic costs [1]. It is the leading cause of chronic kidney disease (CKD) and end-stage renal disease (ESRD). Understanding the underlying causes and risk factors of DN is essential for better management of the disease and its complications [2]. Early detection and treatment of DN can significantly slow, halt, or even reverse the progression to ESRD. The global rise in CKD and ESRD among diabetic individuals is alarming, with India being the second most affected country, reporting 115.1 million CKD cases in 2017. India’s total magnitude and pattern of CKD have been reported sporadically. In urban Indian populations, the prevalence of overt nephropathy and microalbuminuria has been reported as 2.2% and 26.9%, respectively [3].

In the duration of diabetes, systolic blood pressure was the most common risk factor for overt nephropathy and microalbuminuria [4]. DN is characterized by specific pathological, functional, and structural changes in the kidneys of patients with both type 1 and type 2 diabetes, typically presenting as a continuous decline in kidney function, accompanied by persistent albuminuria [5]. Diabetic kidney disease (DKD) is seen in a subset of patients who have both diabetes and CKD, diagnosed by a reduction in the glomerular filtration rate, increased urine albumin excretion, or both [6]. Unlike DN, DKD lacks specific pathological features and may have various other underlying causes. Approximately 30% to 40% of individuals with type 1 or type 2 diabetes develop DKD. The pathogenesis of DN is driven by a complex interaction of metabolic, hemodynamic, growth, and inflammatory or fibrotic factors [7]. Hyperglycemia plays a central role by promoting the formation of advanced glycation end products and activating both receptor-mediated and non-receptor-mediated pathways, leading to tissue damage and organ dysfunction [8]. Moreover, by-products of glucose metabolism can trigger pathways such as the hexosamine, polyol, and protein kinase C pathways, contributing to tissue fibrosis and vascular dysfunction. Increased pressure within the glomeruli affects the tone of afferent and efferent arterioles, while glucose reabsorption in the proximal tubule [9], influenced by insulin, can activate tubule-glomerular feedback, leading to hyperfiltration and subsequent kidney damage [10]. Growth factors, such as vascular endothelial growth factors, can induce hypertensive changes in the kidneys and promote vascular proliferation [11]. Inflammation and fibrosis are key contributors to the development of DN [12]. Trauma or activation of renal cells can trigger the production of chemokines, which in turn activate macrophages [13,14]. These macrophages release cytokines that promote cellular proliferation, ultimately leading to tissue fibrosis [15].

Clinical treatment aims to reduce proteinuria, improve kidney function, and control blood pressure and blood glucose levels [16]. Traditional Indian herbal medicine, including the use of *Cinnamomum tamala* (CT) leaves, has been shown to significantly improve the quality of life for diabetic patients by effectively preventing and treating diabetic nephropathic pain and its complications [17,18]. CT, commonly known as Indian bay leaves, is not only used in cooking but also has a long history in traditional medicine, including Unani and Ayurvedic practices, for treating various ailments [19]. CT exhibits significant cytotoxic effects against certain cancer cell lines. Studies have shown that extracts from CT have potential cytotoxic activity against human glioblastoma cells (U87MG), indicating its promising use in cancer therapy. In vitro tests revealed growth inhibition in these cells, suggesting that compounds from CT could induce cell death in cancerous tissues [20]. In another study, when tested on different cancer cell lines, such as prostate and glioblastoma cells, the bark methanol extract of CT displayed moderate cytotoxic effects [21].

Network pharmacology, a growing field that integrates systems biology and pharmacology, offers a powerful approach for drug discovery and understanding drug action mechanisms. By focusing on the complex interactions between compounds, targets, and diseases, network pharmacology can predict pharmacological effects at both molecular and systemic levels and explore the molecular basis of diseases from a multi-dimensional perspective. In this study, the phytocompounds in CT leaves were analyzed to identify beneficial compounds. Reverse pharmacophore modeling was then employed to identify and validate the target proteins of these compounds. The identified targets were further analyzed through the Kyoto Encyclopedia of Genes and Genomes (KEGG) and Gene Ontology (GO) pathways. Protein–protein interaction (PPI) analysis was used to identify key target proteins, leading to the construction of pharmacological networks that elucidate the mechanisms of treatment. To further investigate, molecular docking was conducted to assess the potential binding of CT compounds with the top 10 most interactive targets involved in DN pathophysiology. The best-docked complexes were subsequently validated through molecular dynamics (MD) simulations and principal component analysis (PCA) to confirm their stability and binding efficacy.

## 2. Materials and Methods

### 2.1. Active Compounds and Correlated Target Database Establishment

To screen the active compounds of CT, the IMPPAT (Indian Medicinal Plants, Phytochemicals, and Therapeutics) database was used (https://cb.imsc.res.in/imppat/home, accessed on 11 April 2024) [22], The plant name “*Cinnamomum tamala*” was entered into the database to retrieve relevant compounds and associated information. The screening criteria for identifying active compounds were based on pharmacokinetics (ADME), with a bioavailability score (BS) of ≥0.5 and drug-likeness (DL) of ≥0.7. The chemical structures and canonical SMILES of the compounds were sourced from PubChem (https://pubchem.ncbi.nlm.nih.gov/, accessed on 11 April 2024) [23]. Using the PharmMapper (https://www.lilab-ecust.cn/pharmmapper/, accessed on 11 April 2024) web tool, the targets associated with CT compounds were identified. PharmMapper is a web server designed to accurately predict the targets of bioactive compounds, with steps taken to remove duplicate targets. The compounds’ SDF format files were obtained and imported into PharmMapper for target prediction, utilizing a reverse pharmacophore matching technique based on structural features [24]. The molecular structures of the active compounds were submitted to PharmMapper, where they were optimized to generate multiple conformations. The molecular structures of the active compounds were submitted to the PharmMapper database, and multiple conformations were generated by optimizing the compound structure. These standardized compound structures were then matched against all human drug targets available in the PharmMapper database, using the UniProt database (https://www.uniprot.org/, accessed on 13 April 2024) as a reference [25]. Targets with a parameter matching score (ft) > 2 were selected as the potential compound targets [26].

### 2.2. Network Construction of Active Compounds–Potential Targets

To illustrate the complex interactions between active compounds and their potential targets, a comprehensive network was constructed using Cytoscape 3.10.1 software (https://www.cytoscape.org/, accessed on 14 April 2024) [27]. In this network, nodes represent the compounds and their corresponding targets, while edges depict the interactions between them.

### 2.3. Collection of Potential DN-Associated Targets

The term “Diabetic nephropathy” was used to search for potential DN targets in the GeneCards (https://www.genecards.org/, accessed on 15 April 2024) database to ensure data accuracy and completeness [28]. Targets with scores higher than the average were selected as potential DN targets, as the GeneCards score reflects the strength of the association between the target and the disease. The target names were then standardized using the UniProt database (https://www.uniprot.org/uploadlists/, accessed on 15 April 2024).

### 2.4. Screening Compound–Disease Overlapping Targets

The filtered CT component targets and DN illness targets were included in the Venn diagram (https://bioinfogp.cnb.csic.es/tools/venny/, accessed on 15 April 2024), and the intersection of the target of the therapeutic ingredient in CT and DN were obtained as potential research goals [25].

### 2.5. Network Construction of Compound–Disease Common Targets

The PPI network, which includes nearly all functional interactions among the identified proteins, was constructed using the STRING platform (https://string-db.org*/*, accessed on 15 April 2024) [29]. The interaction source was set to medium confidence (0.400), and the network type was configured for Homo sapiens [30]. The generated interaction data were then integrated and analyzed using Cytoscape 3.10.1. The CytoHubba plug-in was employed to determine and rank the top ten target proteins based on their interaction scores using the “Degree” method [31].

### 2.6. GO and KEGG Pathway Enrichment Analyses

To accurately characterize the core biological activities within human cells, GO and KEGG pathway analyses were conducted. The enrichment analysis was performed using a bioinformatics platform (http://www.bioinformatics.com.cn, accessed on 16 April 2024) [30]. GO analysis categorized the genes into three primary functional areas: biological processes (BPs), cellular components (CCs), and molecular functions (MFs). KEGG enrichment analysis identified potential targets and biological pathways. Enrichment results were considered significant if they had a *p*-value < 0.05 and an enrichment factor > 1.5. The findings from both GO and KEGG enrichment analyses were visualized using bubble charts [32].

### 2.7. Construction of Compound–Target–Pathway Network

Cytoscape version 3.10.1 was utilized to construct a network illustrating the interactions between compounds, targets, and pathways involved in the mechanism of action of CT active compounds for treating DN [33]. In this network, nodes represent compounds, targets, or disease-related pathways, while edges denote the interactions between these elements.

### 2.8. Molecular Docking

Molecular docking simulations were performed using Schrödinger Maestro (Version 12.8, Release 2021-2, Windows-x64) with extra precision (XP) settings [34]. Proteins were prepared through restrained minimization using the OPLS4 force field. Grid sites for docking were set up with a Glide^®^ receptor grid generator, using a docking box of 20 Å and grid centers determined from the active residues on the target protein [35]. Three-dimensional conformer structures of the essential phytoconstituents were obtained as SDF files from the PubChem database (https://pubchem.ncbi.nlm.nih.gov, accessed on 18 April 2024) [36]. Ligands were prepared using the OPLS4 force field, and their conformations were generated for pH 7.0 ± 2.0. Binding potential between the receptor and ligand was assessed by docking scores (in kcal/mol), with more negative values indicating better binding affinity. A docking score of less than −5 kcal/mol indicated excellent binding activity [37]. The results were visualized using PyMOL 3.0. (https://pymol.org/, accessed on 26 April 2024) [38] and Discovery Studio BIOVIA 2024 (https://discover.3ds.com/, accessed on 26 April 2024) [39] to produce 3D and 2D diagrams of the best-docked target-ligand complexes. The top docking complex was then subjected to MD simulation.

### 2.9. MD Simulation

To assess the stability of the top-docked complex, MD simulations were conducted using Gromacs 2020.4 [40] on a workstation equipped with an Intel Xeon E3-1245 processor (8 cores, 3.50 GHz), 32 GB RAM, and an NVIDIA Quadro P5000 GPU card [41]. The protein topology was prepared using the CHARMM36 all-atom force field [42], and solvation was performed with TIP3P water molecules [43] via the “gmx solvate” command in GROMACS. The ligand topology was generated using the CHARMM General Force Field (CGenFF) [44]. The parameters for bonds, angles, dihedrals, and charges were initially assigned using the CGenFF server provided in the Supporting Information (Table S1). To ensure accuracy, the partial charges were computed using the RESP fitting method [45]. The force field parameters were validated by performing geometry optimization of the ligand using quantum mechanical (QM) calculations [46] and comparing the optimized structure with the force-field-optimized structure. The root mean square deviation (RMSD) between the QM and molecular mechanic (MM)-optimized structures was computed to ensure consistency. Additionally, a potential energy surface (PES) scan was performed for key dihedral angles of the ligand to compare the energy profiles obtained from MM with those from QM calculations [47]. The torsional parameters were adjusted to better match the QM-derived PES. The solvation free energy of the ligand was calculated in water and compared with experimental data to further validate the ligand’s behavior in an aqueous environment [48]. The simulation was set up in a dodecahedron box with the protein–ligand complex centered to ensure a minimum distance of 10 Å from the box boundaries. Sodium chloride (NaCl) was added to a final concentration of 150 mM to replicate physiological conditions [49,50]. The parameters for Na^+^ were as follows: charge = +1.0 e, Lennard-Jones parameters: σ = 2.58 Å, and ε = 0.4184 kJ/mol. For Cl^−^, the parameters were as follows: charge = −1.0 e, Lennard–Jones parameters: σ = 4.40 Å, and ε = 0.4184 kJ/mol [51]. The system underwent energy minimization using the steepest descent method with a maximum of 50,000 steps. Following minimization, equilibration was performed using isothermal–isochoric (NVT) and isothermal–isobaric (NPT) ensembles at 300 K and 1.0 bar pressure, respectively, with temperature and pressure controlled by the Berendsen thermostat [52] and Parrinello–Rahman barostat [53]. A production run of 200 ns was carried out after equilibration, using a 2 fs time step and the Leap-frog integrator [54]. The LINCS algorithm was applied to constrain bonds during NVT, NPT, and production phases [55]. Simulation results were analyzed for RMSD, root mean square fluctuation (RMSF), radius of gyration (Rg), and solvent-accessible surface area (SASA). Each simulation was performed in triplicate, and the results were reported as mean values with standard errors.

### 2.10. Free Energy Calculation (MM-GBSA)

The free energy of protein–ligand complex formation was determined using the Molecular Mechanics/Generalized Born Surface Area (MM/GBSA) method, as previously described [56]. First, the docked complexes were optimized using the MM approach. Next, energy minimization was carried out with the OPLS4 force field in conjunction with the Generalized Born Surface Area (GBSA) continuum solvent model. The binding free energies of the protein–ligand complexes were then calculated using the following equations:

∆G_Bind_ = ∆E_MM_ + ∆G_Solv_GB_ + ∆G_SA_

where ∆G_Bind_, ∆E_MM_, ∆G_(Solv_GB)_, and ∆G_SA_ are the binding free energy between a ligand and its target, molecular mechanical energy, solvation free energy calculated using the Generalized Born (GB) model, and non-polar solvation free energy, respectively.

∆E_MM_ = Ec_omplex_
*−* (E_protein_ + E_ligand_)

where E_ligand_, E_protein_, and Ec_omplex_ are the minimized energies of the ligand, protein, and protein–ligand complex, respectively.

∆G_solv_GB_ = G_solvGB(complex)_ − G_solvGB(protein)_ + G_solv_GB(ligand)_

where G_solv_GB(ligand)_, G_solvGB(protein)_, and G_solvGB(complex)_ are the free energies of solvation of the ligand, protein, and protein–ligand complex, respectively.

∆G_SA_ = G_SA(compelx)_ − G_SA(protein)_ + G_SA(ligand)_

where G_SA (Ligand)_, G_SA (Protein)_, and G_SA (Complex)_ are the surface area energies of the ligand, protein, and protein–ligand complex, respectively. The free energy, in the Prime-MM/GBSA method, is calculated as follows:

∆G_Bind_ = ∆G_Coulomb_ + ∆G_vdW_ + ∆G_Covalent_ + ∆G_H−bond_ + ∆G_Sol_Lipo_ + ∆G_Solv_GB_ + ∆G_Packing_ + ∆G_Self−contact_

where ∆G_Coulomb_, ∆G_vdW_, ∆G_Covalent_, ∆G_H−bond_, ∆G_Sol_Lipo_, ∆G_Solv_GB_, ∆G_Packing_, and ∆G_Self−contact_ are the Coulombic (electrostatic) contribution, van der Waals contribution, hydrogen bond contribution to the binding free energy between the ligand and its receptor, lipophilic (hydrophobic) contribution to solvation free energy, polar solvation free energy by the GB model, packing free energy contribution, and self-contact free energy or self-energy of a molecule.

### 2.11. PCA

The collective motion of the protein and its ligand was analyzed using PCA with the Bio3D package [57]. Initially, translational and rotational motions of the protein were ignored. The covariance matrix and its eigenvectors were then calculated by superimposing the atomic coordinates of the protein onto a reference structure. The symmetric covariance matrix was diagonalized using an orthogonal transformation matrix, resulting in a diagonalized matrix of eigenvalues. The covariance matrix (C) was computed using the following equation:

(1) $$C_{ij}=\langle \left(x_{i}-\langle x_{i}\rangle \right)\left(x_{j}-\langle x_{j}\rangle \right)\rangle \;\;\;\;i,j=1,2,3,\ldots ..,3N$$

where, N, x_i/j_, and <x_i/j_> represent the number of Cα-atoms, the Cartesian coordinate of the ith/jth Cα-atom, and time average of all the conformations, respectively.

## 3. Results

### 3.1. Active Compounds and Correlated Target Network

The IMPPAT database was utilized to identify active phytochemical compounds from CT. Six compounds were selected for further investigation based on BS ≥ 0.5 and DL ≥ 0.7 (Table 1). A compound–target network was then constructed to illustrate the interactions between the CT compounds and their respective targets (Figure S1). This network included six compounds mapped to 347 potential targets, resulting in 353 nodes and 1217 edges. In the network, blue round rectangles represent the putative targets, while pink diamond shapes denote the CT compounds. The selected compounds—Cubebol, Methoxyeugenol, Benzyl benzoate, Isoeugenol, Elemol, and beta-Bisabolol—correspond to 189, 271, 168, 197, 153, and 239 targets, respectively. These findings suggest that these six compounds may play significant therapeutic roles in treating DN.

### 3.2. Screening of Common Targets

**Figure 1:**
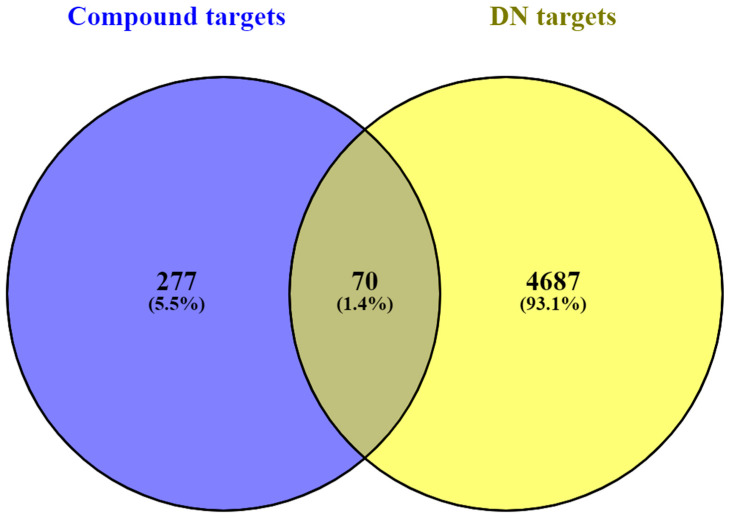
Venn diagram illustrating the overlap between DN targets and targets associated with CT compounds. The blue and yellow sections represent targets from the CT compounds and those obtained from the GeneCards database, respectively.

By excluding the CT-connected targets from the GeneCards database, the results were consolidated. A total of 4757 potential target genes were collected from GeneCards for further research. Figure 1 illustrates 70 potential target genes identified through Venn diagram analysis, which was performed by intersecting 4687 DN-related targets with 347 potential targets of CT active compounds.

### 3.3. PPI Network Analysis

**Figure 2:**
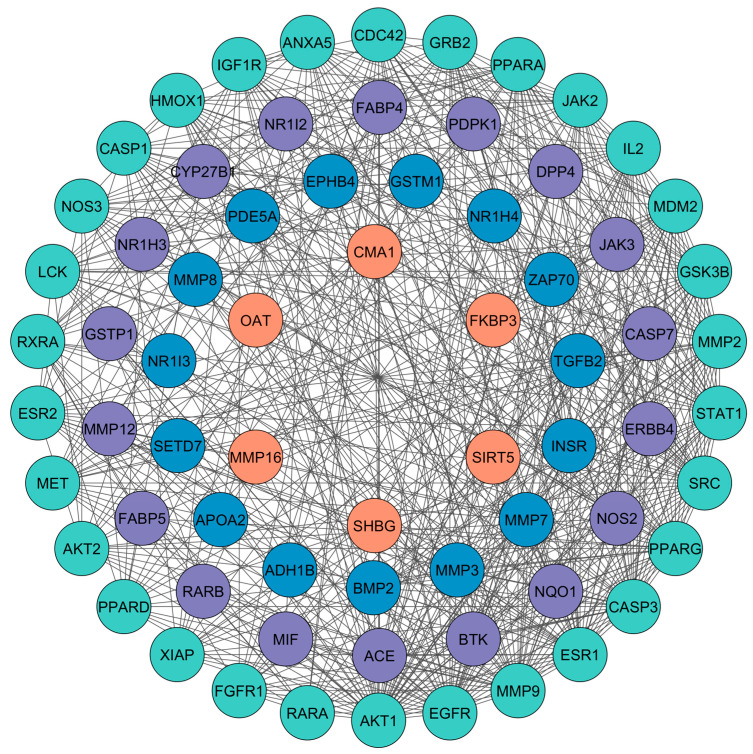
PPI network obtained from Cytoscape software. Circles represent nodes, and edges represent the relation between nodes.

**Figure 3:**
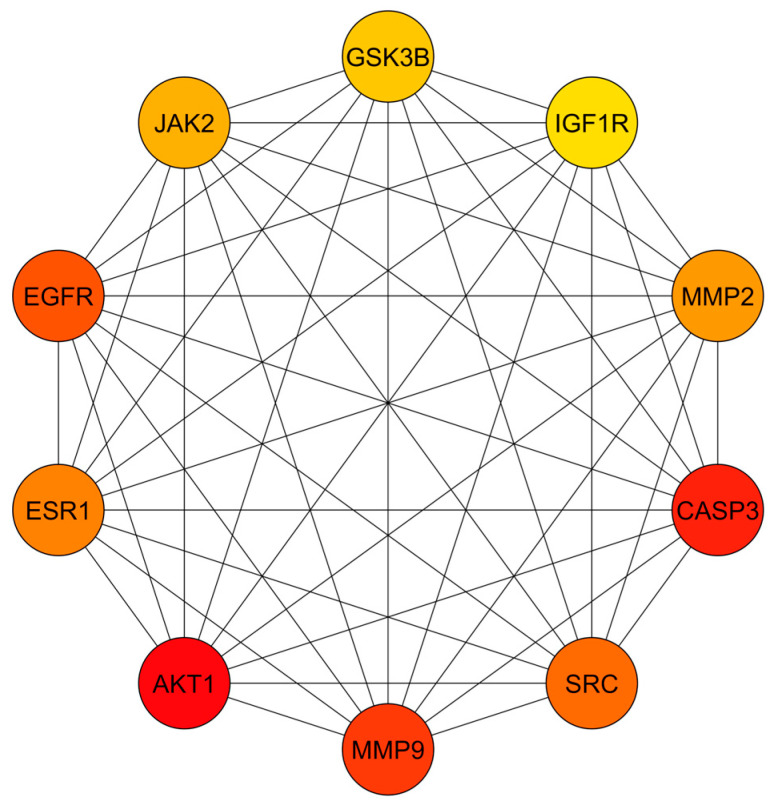
Core PPI network for top 10 targets obtained from CytoHubba plug-in Cytoscape software by MCC method. Circles represent nodes, and edges represent the relation between nodes.

For PPI network analysis, the 70 predicted targets were imported into STRING, resulting in a network comprising 70 nodes and 562 edges, with an average node degree of 16.1 (Figure S2). The network was constructed with a medium confidence score of 0.400, incorporating all relevant interaction sources. The PPI enrichment *p*-value was <1.0 × 10^−16^. The resulting data were imported into Cytoscape for further visualization and analysis (Figure 2). In this network diagram, nodes represent the targets, edges illustrate interactions between targets, and edge thickness indicates the strength of these interactions. To identify the core PPI network, CytoHubba was utilized with the MCC method, which highlighted the top 10 nodes with the most significant centrality. These core targets, listed in descending order, are MMP9, EGFR, AKT1, IGF1R, JAK2, MMP2, SRC, ESR1, CASP3, and GSK3B (Figure 3). The complete set of 70 targets was also used for pathway enrichment analysis. These primary targets were then incorporated into the compound–target–pathway network and evaluated through molecular docking studies to assess their potential efficacy in treating DN.

### 3.4. GO and KEGG Enrichment Analyses

**Figure 4:**
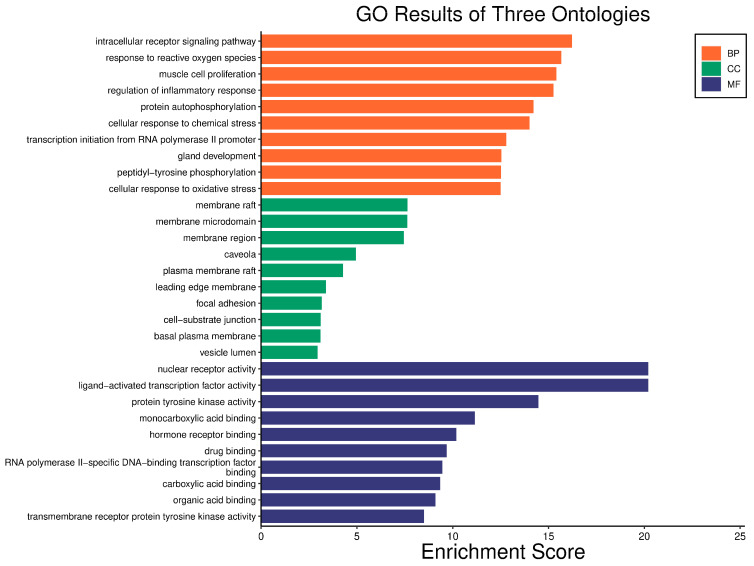
Visualization of top 10 BPs, CCs, and MFs in bar-plot diagram (The pathway is shorted by Fold enrichment, and the x-axis shows −logP).

**Figure 5:**
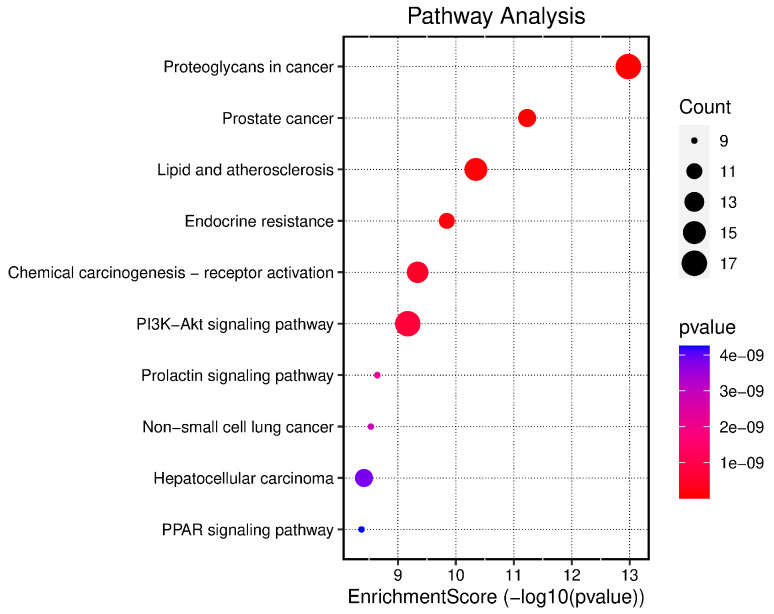
Visualization of top 10 KEGG pathways in a dot-plot diagram (The pathway is shorted by Fold enrichment, dots show the gene count number, the x-axis shows −logP, and the color shows the *p*-value).

Gene products can be critically annotated using GO assessment. The impact and mechanisms of protein transport in biological pathways were highlighted through BP enrichment. The CC analysis indicated the involvement of proteins in various cellular environments. MF analysis demonstrated the functions of specific protein receptors modulated by pharmacological agents. Using the Bioinformatics platform, GO functional enrichment analysis was performed on the 70 intersection targets related to DN and the active phytochemical compounds used in its treatment, focusing on BP, CC, and MF categories (Figure 4). From the 3085 BP items, the top 10 were selected for visualization, including intracellular receptor signaling pathways, response to reactive oxygen species, and muscle cell proliferation. Among the 154 CC items, the top 10 were chosen, highlighting membrane rafts, membrane microdomains, and membrane regions. Additionally, from the 320 MF items, the top 10 entries included nuclear receptor activity, ligand-activated transcription factor activity, and protein tyrosine kinase activity. Based on enrichment factor values and the number of genes involved in each pathway, 10 signaling pathways with high relevance to DN mechanisms were identified, as shown by the log *p*-values in Figure 5. The related information is detailed in Table 2. The color scale (blue to red) represents the size of the log *p*-value for each pathway, while the circle size indicates the number of associated targets.

### 3.5. Compound–Target–Pathway Network Construction

**Figure 6:**
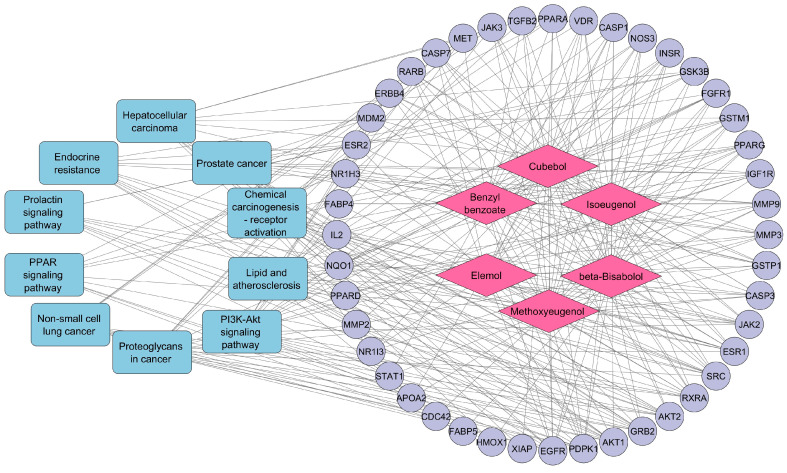
Compound–target–pathway network constructed by Cytoscape software (blue round rectangle nodes represent pathway names, pink diamond-shaped nodes represent compounds, purple circular-shaped nodes represent targets, and edges represent the relation between nodes).

Target pathway data from KEGG analysis were imported into Cytoscape software to construct a network graph illustrating the relationships between compounds, targets, and pathways. This visualization aids in understanding the involvement of each compound with specific targets and the pathways each target is associated with. Figure 6 shows the compound–target–pathway network for CT, featuring 60 nodes (six compounds, 44 targets, and 10 pathways) and 298 edges. The network analysis indicates that multiple CT compounds interact with at least ten targets. Additionally, most target genes are regulated by one or more active compounds, with many potentially involved in DN-related pathways. This network analysis highlights the roles of various CT compounds and targets in the treatment of DN.

### 3.6. Molecular Docking of Compounds and Targets

**Figure 7:**
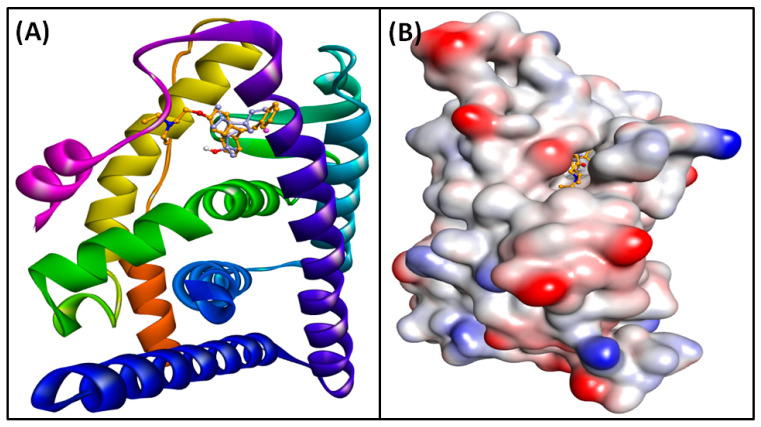
The docked complex of control Clomifene (yellow compound) and Elemol (blue compound) with the receptor ESR1. ((**A**): Protein–ligand complex and (**B**): mesh diagram of protein–ligand complex.)

**Figure 8:**
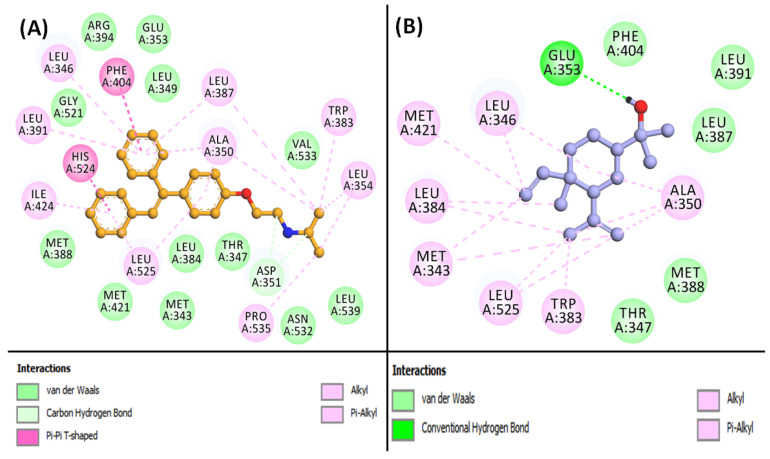
The binding site overview of the docked complex of [(**A**): control Clomifene (yellow compound) and (**B**): Elemol (blue compound)] with the receptor ESR1.

To validate the findings from network pharmacology, molecular docking was employed to evaluate the interactions between screened active compounds and key targets. The active compounds and principal targets identified in the core PPI network were subjected to docking studies. The absorption, distribution, metabolism, and excretion (ADME) properties of the six selected compounds were assessed using the SwissADME tool (http://www.swissadme.ch/, accessed on 18 April 2024). The PDB IDs for the selected targets were AKT1: 3O96, CASP3: 3KJF, EGFR: 5UGB, ESR1: 6VPF, GSK3B: 4ACC, IGF1R: 2OJ9, JAK2: 4IVA, MMP2: 3AYU, MMP9: 1GKC, and SRC: 2BDJ. Table 3 lists the free binding energies (kcal/mol) for the key targets and active compounds obtained from molecular docking. Among the ten targets, AKT1 and ESR1 exhibited strong binding affinities with all compounds (≤−5 kcal/mol). Elemol demonstrated the highest binding affinity to ESR1 with a binding energy of −8.674 kcal/mol, while Benzyl benzoate showed the weakest binding, with EGFR at 0.099 kcal/mol. Other compounds such as beta-bisabolol, Cubebol, Elemol, and Methoxyeugenol also had significant binding affinities for ESR1. Isoeugenol had the highest binding affinity for JAK2. Cubebol displayed the best overall binding affinity with AKT1 (−7.449 kcal/mol), while Methoxyeugenol had strong binding with CASP3 (−5.118 kcal/mol), Isoeugenol had strong binding with EGFR (−5.323 kcal/mol), and Benzyl benzoate had strong binding with IGF1R (−7.167 kcal/mol), MMP9 (−5.624 kcal/mol), and SRC (−6.497 kcal/mol). These results suggest that CT compounds may be effective in treating DN. Elemol, in particular, showed the best docking results with ESR1 compared to the control inhibitor Clomifene. Elemol was able to occupy the central cavity of ESR1, a site also occupied by Clomifene (Figure 7A and Figure 8B). Clomifene’s docking energy with ESR1 was −10.2 kcal/mol. Clomifene interacted with ESR1 through two carbon–hydrogen bonds with ASP351 (Figure 8A, Table S2) and was stabilized by sixteen hydrophobic interactions with residues such as LEU346, ALA350, LEU354, TRP383, LEU387, LEU391, PHE404, LEU424, HIS524, LEU525, and PRO535. Additionally, Clomifene formed van der Waals interactions with residues including MET343, THR347, LEU349, GLU353, LEU384, MET388, ARG394, MET421, GLY521, ASN532, and VAL533. By contrast, the Elemol–ESR1 complex was stabilized by one conventional hydrogen bond with GLU353 (Figure 8B, Table S2). Elemol also formed thirteen hydrophobic interactions with LEU326, MET343, ALA350, TRP383, LEU384, MET421, and LEU525. Furthermore, Elemol engaged in van der Waals interactions with THR347, LEU387, MET388, LEU391, and PHE404. Given the promising results, this complex was selected for MD simulation analysis.

### 3.7. Analysis of MD Simulation

MD simulation is a vital computational technique in drug discovery, providing detailed insights into the dynamic behavior of biological macromolecules and their interactions with potential drug candidates. By examining structural changes, binding affinities, and conformational fluctuations in protein–ligand complexes, MD simulations facilitate rational drug design and virtual screening. This approach helps accelerate the development of new therapeutics with improved efficacy and safety profiles.

#### 3.7.1. RMSD Analysis

**Figure 9:**
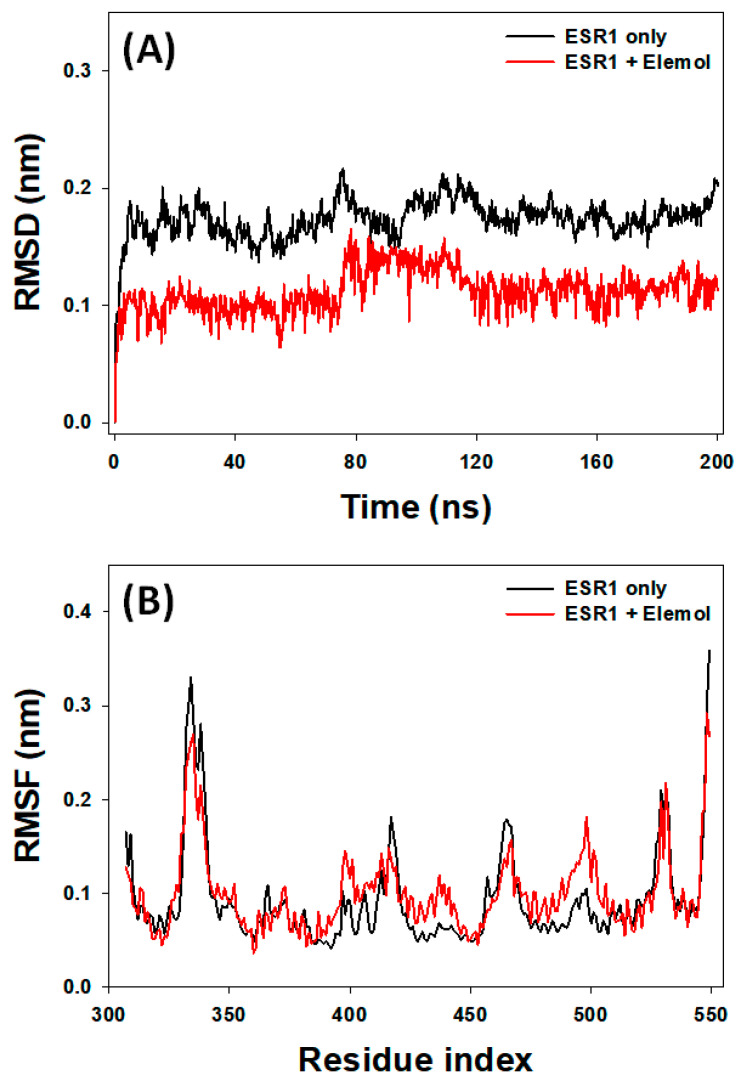
Graph plot ((**A**): RMSD and (**B**): RMSF).

RMSD is a critical measure for evaluating the stability of protein–ligand complexes during MD simulations, as it quantifies deviations of the structure from its initial state throughout the simulation period [58]. In this study, RMSD (Cα-atoms) for ESR1 alone and the ESR1–Elemol complex was analyzed over a 200 ns simulation period. For ESR1 alone, RMSD showed fluctuations during the initial 5 ns before stabilizing. The RMSD of ESR1 alone ranged from 0.137 to 0.217 nm, with an average value of 0.175 ± 0.008 nm from 5 to 200 ns (Figure 9A). Similarly, the RMSD of the ESR1–Elemol complex varied during the first 5 ns but stabilized as favorable interactions between the protein and ligand developed. Throughout the 5 to 200 ns period, the RMSD of the ESR1–Elemol complex ranged from 0.064 to 0.166 nm, with an average of 0.112 ± 0.006 nm.

#### 3.7.2. RMSF Analysis

RMSF analysis is crucial for understanding how ligand binding impacts the conformational dynamics of amino acid residues within proteins [56]. In this study, RMSF was used to examine changes in the side-chain conformations of amino acid residues in both ESR1 alone and the ESR1–Elemol complex. The RMSF plot identifies peaks that represent regions of increased flexibility, typically found in protein loops and terminal ends. The RMSF profile for the ESR1–Elemol complex was similar to that of ESR1 alone (Figure 9B), indicating minimal changes in ESR1’s conformation upon Elemol binding. Nonetheless, subtle differences were observed, likely due to Elemol’s insertion and positioning within ESR1’s binding site. Overall, RMSF analysis highlights the dynamic behavior of ESR1 in the presence of Elemol, providing insights into the structural changes induced by ligand binding and enhancing our understanding of the molecular interactions between ESR1 and Elemol.

#### 3.7.3. Rg Analysis

**Figure 10:**
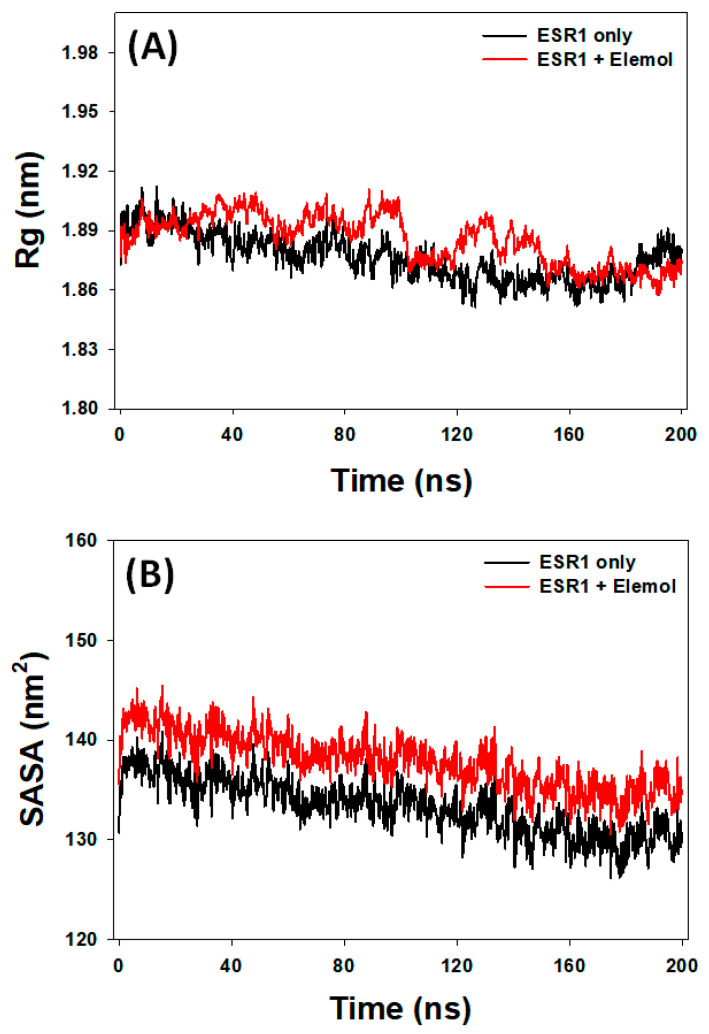
Graph plot ((**A**): Rg and (**B**): SASA).

Rg measurements are instrumental in evaluating key aspects of ligand–protein interactions, such as ligand occupancy within the binding site and the overall compactness of the protein–ligand complex [59]. In this study, Rg was used to assess ESR1’s behavior both in isolation and in a complex with Elemol. Over the 0 to 200 nanosecond simulation period, the Rg values for ESR1 ranged from 1.851 to 1.912 nm without Elemol and from 1.868 to 1.914 nm with Elemol (Figure 10A). The average Rg values were 1.880 ± 0.005 nm for ESR1 alone and 1.895 ± 0.007 nm for the ESR1–Elemol complex. These results indicate that Elemol slightly increases the compactness of ESR1, suggesting stable packing of the ESR1–Elemol complex throughout the simulation. This finding underscores Elemol’s potential role in modulating ESR1’s structural dynamics, which could influence its functional activity as a ligand.

#### 3.7.4. SASA Analysis

SASA are a valuable technique for assessing the exposure of a ligand within a protein’s binding site and its interactions with surrounding water molecules [60]. This study applied SASA to analyze ESR1’s behavior with and without Elemol. During the simulation period from 0 to 200 nanoseconds, ESR1’s SASA values ranged from 126.1 to 140.9 nm^2^ without Elemol and from 130.5 to 145.5 nm^2^ with Elemol (Figure 10B). The average SASA values were 133.2 ± 0.7 nm^2^ for ESR1 alone and 137.9 ± 0.8 nm^2^ for the ESR1–Elemol complex. These results suggest that Elemol may slightly impact the surface accessibility of ESR1, potentially indicating changes in the protein’s binding site conformation or its interactions with solvent molecules.

#### 3.7.5. Analysis of Hydrogen Bonds

**Figure 11:**
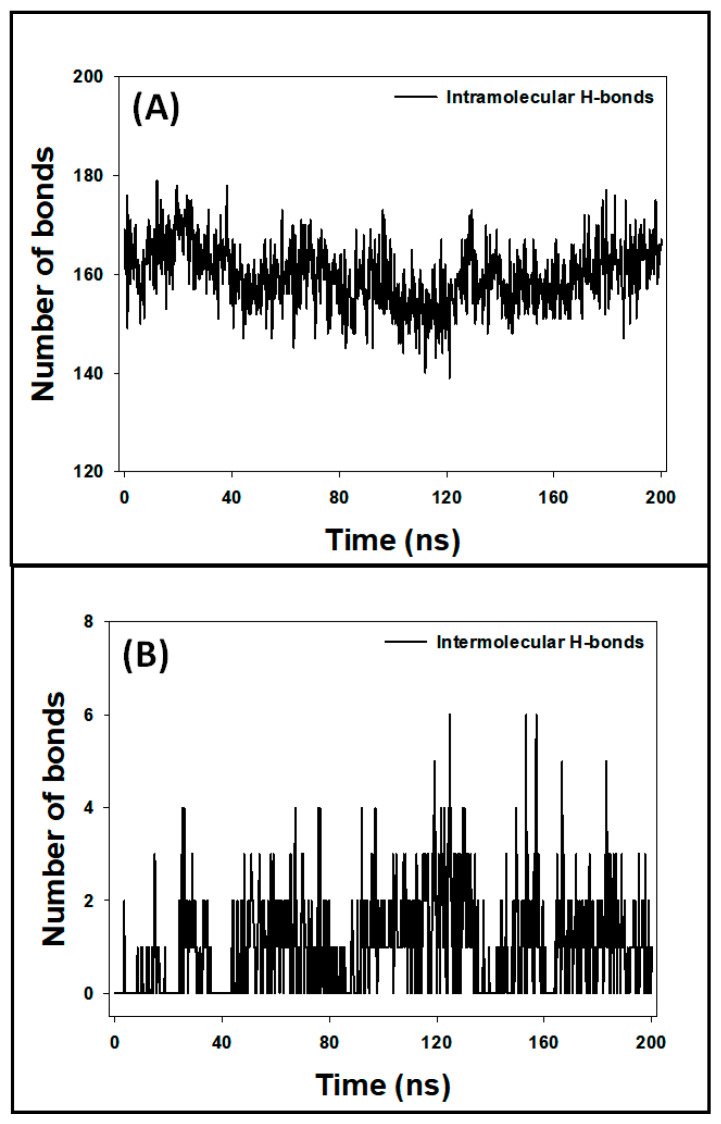
Graph plot ((**A**): intramolecular hydrogen bonds within the ESR1 protein and (**B**): intramolecular hydrogen bond of ESR1 protein with Elemol).

During MD simulation, assessing the stability of the protein–ligand complex is essential. This can be achieved by analyzing hydrogen bonds within the protein (intramolecular) and between the protein and the ligand (intermolecular) throughout the simulation. In this study, the number of intramolecular hydrogen bonds within the ESR1 protein fluctuated between 139 and 179 (Figure 11A), reflecting the dynamic nature of the protein’s structure. This indicates the dynamic nature of the protein’s structure during the simulation. Meanwhile, the intermolecular hydrogen bonds formed between the ESR1 protein and the ligand, Elemol, were observed to fluctuate between 0 and 6 bonds (Figure 11B). Despite these fluctuations, the presence of consistent hydrogen bonds between ESR1and Elemol throughout the MD simulation suggests that the complex remains stable over time. The formation of stable hydrogen bonds between the protein and the ligand is indicative of favorable interactions between them. These interactions are essential for maintaining the structural integrity of the protein–ligand complex and may contribute to the overall effectiveness of the ligand in modulating the protein’s function. Therefore, the observed stability of the ESR1–Elemol complex implies that Elemol could potentially act as a reliable ligand for ESR1 under physiological conditions.

### 3.8. Analysis of Free Energy Calculations (MM/GBSA)

To assess the binding strength of ESR1 with Elemol, the free energy, representing the protein–ligand interactions in a solvent environment, was calculated using the MM/GBSA method. The results, shown in Table 4, reveal that the ESR1–Elemol complex had free energy (−49.85 kcal/mol). In this context, the primary contributors to the stability of the protein–ligand complexes were Coulombic energy (∆G_Coulomb_), lipophilic energy (∆G_SA_ or ∆G_Sol_Lipo_), and van der Waals energy (∆G_vdW_). By contrast, the polar solvation energy (∆G_Solv_ or ∆G_SolGB_) and covalent energy (∆G_Covalent_) were the main forces opposing the protein–ligand interactions. These findings are consistent with molecular docking results, highlighting that Elemol exhibits significant inhibitory potential against ESR1 (Table 4).

### 3.9. PCA

**Figure 12:**
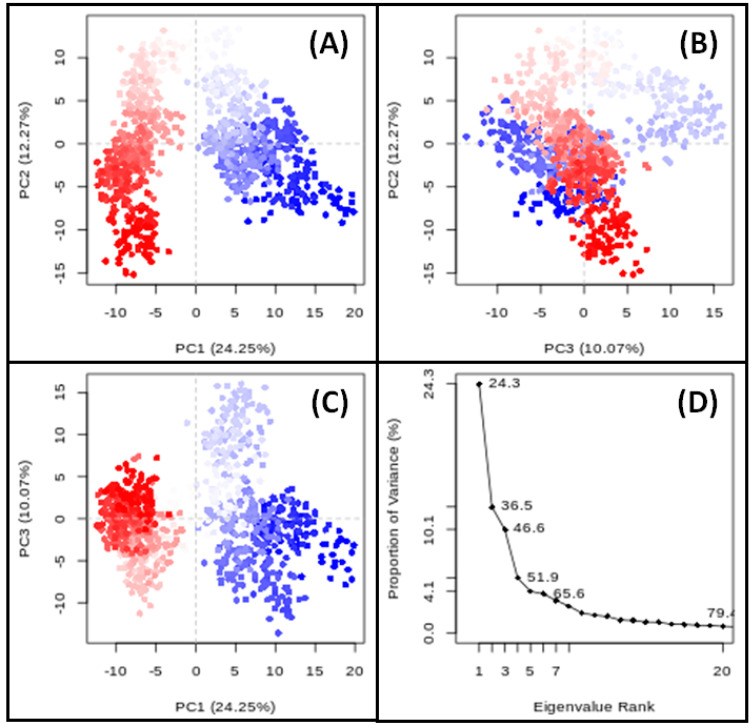
PCA of ESR1 in the presence of Elemol ((**A**): PC1−PC2 projection, (**B**): PC2−PC3 projection, (**C**): PC1−PC3 projection, (**D**): the conformational variances).

PCA is a valuable tool for evaluating target proteins’ motion and conformational changes during simulations. This study employed PCA to investigate the conformational dynamics of ESR1 in a complex with Elemol along the PC1–PC2, PC2–PC3, and PC1–PC3 axes, based on Cα-atoms (Figure 12). Each red and blue dot on the plots represents a distinct conformational state of ESR1, with the clusters indicating energetically favorable regions of conformational space. For the PC1–PC2 projection, the ESR1–Elemol complex occupied a conformational subspace ranging from −12 to +20 along PC1 (24.25% variance) and from −16 to +20 along PC2 (12.27% variance) (Figure 12A). In the PC2–PC3 projection, the conformational space spanned from −16 to +20 along PC2 (12.27% variance) and from −15 to +16 along PC3 (10.07% variance) (Figure 12B). The PC1–PC3 projection showed a range from −12 to +20 along PC1 (24.25% variance) and from −15 to +16 along PC3 (10.07% variance) (Figure 12C). Overall, the first three principal components of ESR1 in the presence of Elemol accounted for 46.6% of the total conformational variance (Figure 12D). This indicates that these principal components are crucial for understanding the structural dynamics of ESR1 induced by Elemol binding.

## 4. Discussion

**Figure 13:**
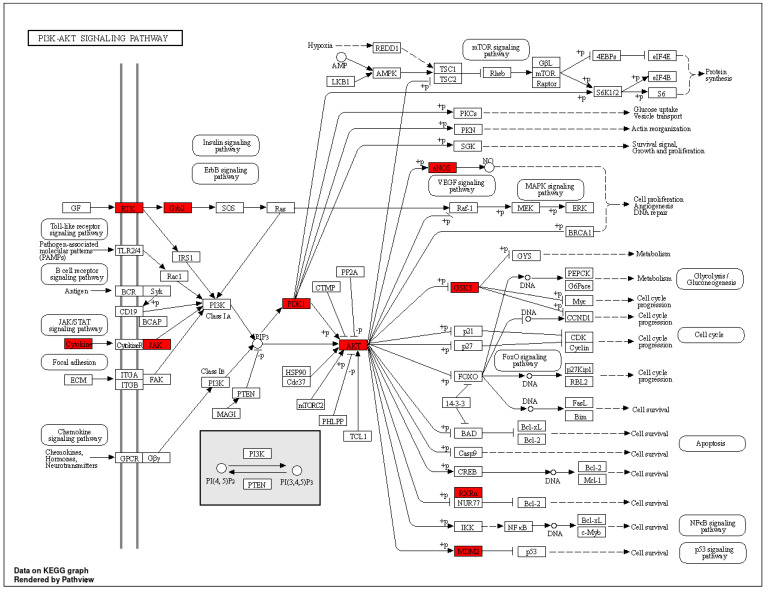
KEGG pathway diagram for PI3K−AKT signaling pathway(The targets for the selected compounds are highlighted in red).

**Figure 14:**
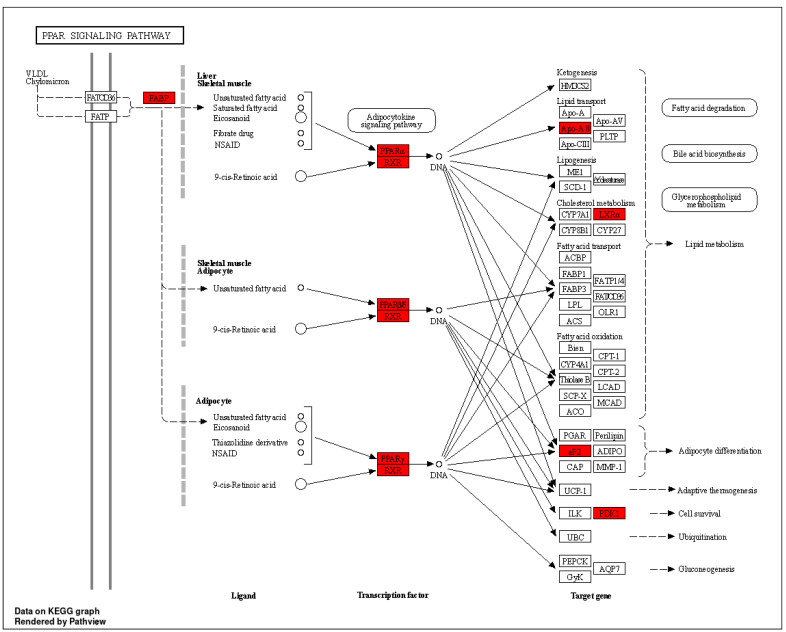
KEGG pathway diagram for PPAR signaling pathway (The targets for the selected compounds are highlighted in red).

By 2030, diabetes is projected to affect over 360 million people globally, representing a major public health issue [61]. Diabetes is characterized by abnormal blood glucose levels and complications such as endothelial damage, cardiomyopathy, retinopathy, atherosclerosis, erectile dysfunction, nephropathy, and neuropathy [62]. Among these, DN remains a critical concern despite existing treatments [63]. This underscores the need for safer and more effective therapies. Research supports the potential of herbal medicine for treating DN, highlighting its effectiveness [18]. Traditional Indian medical systems, such as Ayurveda and Unani, have long used herbal remedies for disease prevention and treatment, including diabetes-related conditions [64,65]. CT, known as *tejpat* and *tejpatra* in Sanskrit, has demonstrated hypolipidemic and hypoglycemic properties. It is rich in phenolic compounds with antioxidant activities that may affect multiple targets and pathways related to DN. Prior research has shown the nephroprotective effects of CT extracts against Gentamicin-induced nephrotoxicity in rabbits [66]. Six active compounds from CT were selected for this study based on BS and DL, including Benzyl benzoate, beta-Bisabolol, Cubebol, Elemol, Isoeugenol, and Methoxyeugenol. This work used network pharmacology and molecular docking approaches to comprehend the molecular biology of CT for the treatment of DN. Six CT compounds suggested 347 potential targets using PharmMapper, and 4687DN-related targets were acquired from the GeneCards database. The Venn diagram eliminated 70 potential CT and DN common targets. According to the compound–target–pathway network analysis, all of the compounds in CT have the potential to act on various targets and follow associated pathways for the treatment of DN. To perform GO enrichment and predict the mechanism of CT in treating DN, 70 common targets between the compounds of CT and DN were chosen. These objectives included BPs, CCs, and MFs. We found that the candidate targets are involved in multiple BPs, such as intracellular receptor signaling pathways, response to reactive oxygen species, and muscle cell proliferation [67,68]. The active targets such as AKT1, JAK2, EGFR, ESR1, and SRC mainly participate in the BPs. It was discovered that the potential targets are connected to many CCs, including membrane raft, membrane microdomain, and membrane region [69]. CT compounds may have a role in controlling these CCs, thereby reducing the severity of DN. Moreover, DN is directly associated with nuclear receptor activity, ligand-activated transcription factor activity, and protein tyrosine kinase activity, which are mainly enriched in MFs [70,71,72]. Thus, the MFs of several targets are relatively consistent with the pathogenesis and mechanism of clinical DN. Using network pharmacological analysis and performing KEGG enrichment, we found that these CT compounds may relieve the symptoms of DN through the action of targets in various signaling pathways and multiple BPs, including the PI3K-Akt signaling pathway (Figure 13) [73], PPAR signaling pathway (Figure 14) [74], hepatocellular carcinoma [75], prolactin signaling pathway [76] and non-small-cell lung cancer [77], which are associated with the development of DN and evoke oxidative stress and chronic inflammation in renal tissues, ending up in losses in kidney function by activating various intracellular signaling pathways like PI3K/Akt/mTOR, EGFR, JAK2 and ESR1. Additionally, it is believed that reactive oxygen species can regulate PI3K/Akt/mTOR signaling and play an essential role in the development of DN, including epithelial–mesenchymal transition [78]. During EMT, epithelial cells lose their primary epithelial properties, such as epithelial- (E-) cadherin, while acquiring characteristics typical of mesenchymal cells such as α-SMA, ending up with renal interstitial fibrosis. Moreover, PI3K/Akt/mTOR signaling can promote high glucose-induced podocyte apoptosis, which contributes to the pathogenesis of DN [79]. Down-regulation of Akt activation during long-term hyperglycemia contributes to enhanced p38 MAPK activation and RPTC apoptosis, and excessive activation of the PI3K/Akt signaling pathway can promote the development and progression of DN [80,81]. From the molecular docking analysis, all the active compounds showed good binding affinity, validating their having a potential role in controlling DN. Activation of the JAK/STAT signaling cascade, including JAK2 as a potential activator, can stimulate excessive proliferation and growth of glomerular mesangial cells, contributing to DN [82]. Isoeugenol, Methoxyeugenol, Cubebol, and Benzyl benzoate exhibited strong binding affinity for JAK2, suggesting their potential effectiveness in treating DN. The compound–target–pathway network analysis indicated that all six active compounds from CT interact with pathways involved in DN through multiple targets. This suggests that these compounds could potentially treat DN by influencing various pathways and targets. Molecular docking results were further validated by MD simulation and PCA of the ESR1–Elemol complex. However, further validation through *in vitro* and *in vivo* studies is necessary to confirm these findings. This study’s accuracy and reliability were dependent on the quality of data extracted from the literature and databases, highlighting the need for further analysis of CT’s active ingredients using techniques such as LC/MS. Additionally, clinical trials and animal studies are required to substantiate the predictive findings of this research.

## 5. Conclusions

This research is the first to explore the pharmacological and molecular mechanisms of CT for treating DN using a combination of bioinformatics tools, including network pharmacology, molecular docking, MD simulation, and PCA. We identified six active compounds in CT and compiled a list of 347 targets related to these compounds, as well as 4687 targets associated with DN. PPI analysis revealed ten significant targets, with key signaling pathways such as the PI3K-AKT and PPAR pathways playing crucial roles in CT’s effects on DN. Network pharmacology analysis indicated that CT might exert its therapeutic effects by modulating these pathways. Significant target proteins identified include AKT1, JAK2, EGFR, ESR1, and SRC. This study highlights the multi-target and multi-pathway nature of CT’s active compounds and their potential mechanisms of action. The findings provide valuable insights into CT’s role in managing DN and suggest directions for future research. However, due to the reliance on network databases and molecular docking, additional *in vivo* and *in vitro* studies are needed to validate these results and confirm the precise therapeutic mechanisms of CT.

## Figures and Tables

**Table 1:** General and pharmacokinetic information of phytoconstituents of CT. (TPSA: Total polar surface area.)

COMPOUND	CID	SMILES	BS	DL	Molecular Weight (g/mol)	Num. Heavy Atoms	TPSA(Å^2^)	GI Absorption
Cubebol	11276107	CC1CCC(C2C13C2C(CC3)(C)O)C(C)C	0.55	0.72	222.37	16	20.23	High
Methoxyeugenol	226486	COC1=CC(=CC(=C1O)OC)CC=C	0.55	0.75	194.23	14	38.69	High
Benzyl benzoate	2345	C1=CC=C(C=C1)COC(=O)C2=CC=CC=C2	0.55	0.73	212.24	16	26.30	High
Isoeugenol	853433	CC=CC1=CC(=C(C=C1)O)OC	0.55	0.73	164.20	12	29.46	High
Elemol	92138	CC(=C)C1CC(CCC1(C)C=C)C(C)(C)O	0.55	0.72	222.37	16	20.23	High
beta-Bisabolol	12300146	CC1=CCC(CC1)(C(C)CCC=C(C)C)O	0.55	0.71	222.37	16	20.23	High

**Table 2:** Top 10 KEGG pathway-related information.

Pathway ID	Description	*p*-Value	Gene IDs	Gene Count
hsa05205	Proteoglycans in cancer	1.05552 × 10^−13^	SRC/FGFR1/PDPK1/EGFR/ESR1/MET/IGF1R/MMP9/TGFB2/CASP3/MDM2/ERBB4/AKT2/MMP2/GRB2/AKT1/CDC42	17
hsa05215	Prostate cancer	5.88521 × 10^−12^	MMP3/FGFR1/PDPK1/EGFR/IGF1R/GSTP1/MMP9/GSK3B/MDM2/AKT2/GRB2/AKT1	12
hsa05417	Lipid and atherosclerosis	4.51366 × 10^−11^	MMP3/SRC/PDPK1/RXRA/PPARG/MMP9/CASP3/CASP1/CASP7/NOS3/GSK3B/JAK2/AKT2/AKT1/CDC42	15
hsa01522	Endocrine resistance	1.43526 × 10^−10^	SRC/EGFR/ESR1/IGF1R/MMP9/ESR2/MDM2/AKT2/MMP2/GRB2/AKT1	11
hsa05207	Chemical carcinogenesis—receptor activation	4.57818 × 10^−10^	SRC/EGFR/RXRA/PPARA/ESR1/VDR/GSTM1/ESR2/JAK2/NR1I3/AKT2/GRB2/XIAP/AKT1	14
hsa04151	PI3K-Akt signaling pathway	6.77448 × 10^−10^	FGFR1/PDPK1/EGFR/RXRA/MET/IGF1R/IL2/INSR/NOS3/GSK3B/MDM2/ERBB4/JAK2/JAK3/AKT2/GRB2/AKT1	17
hsa04917	Prolactin signaling pathway	2.27113 × 10^−9^	SRC/ESR1/STAT1/ESR2/GSK3B/JAK2/AKT2/GRB2/AKT1	9
hsa05223	Non-small cell lung cancer	2.93718 × 10^−9^	PDPK1/EGFR/RXRA/MET/JAK3/AKT2/RARB/GRB2/AKT1	9
hsa05225	Hepatocellular carcinoma	3.85957 × 10^−9^	EGFR/MET/IGF1R/GSTP1/NQO1/TGFB2/GSTM1/GSK3B/AKT2/HMOX1/GRB2/AKT1	12
hsa03320	PPAR signaling pathway	4.25749 × 10^−9^	PDPK1/RXRA/PPARA/PPARG/FABP4/NR1H3/APOA2/FABP5/PPARD	9

**Table 3:** Binding affinity (Kcal/mol) and ligand efficiency (on bracket) information on targets and compounds from molecular docking.

	Benzyl Benzoate (2345)	Beta-Bisabolol (12300146)	Cubebol (11276107)	Elemol (92138)	Isoeugenol (853433)	Methoxyeugenol (226486)
AKT1	−6.404 (−0.400)	−6.753 (−0.466)	−7.449 (−0.466)	−6.217 (−0.389)	−6.113 (−0.509)	−6.141 (−0.439)
CASP3	−1.893 (−0.118)	−2.449 (−0.153)	−3.448 (−0.216)	−2.100 (−0.131)	−3.969 (−0.331)	−5.118 (−0.366)
EGFR	0.099 (0.006)	−4.415 (−0.276)	−3.851 (−0.241)	−4.041 (−0.253)	−5.323 (−0.444)	−5.268 (−0.376)
ESR1	−8.199 (−0.512)	−7.686 (−0.480)	−8.413 (−0.526)	−8.674 (−0.542)	−6.709 (−0.559)	−6.964 (−0.497)
GSK3B	−2.490 (−0.156)	−2.483 (−0.155)	−2.589 (−0.162)	−2.198 (−0.137)	−2.887 (−0.241)	−3.233 (−0.231)
IGF1R	−7.167 (−0.448)	−4.553 (−0.285)	−4.930 (−0.308)	−4.005 (−0.250)	−5.430 (−0.453)	−6.158 (−0.440)
JAK2	−6.092 (−0.381)	−3.758 (−0.235)	−5.009 (−0.313)	−4.302 (−0.269)	−6.800 (−0.567)	−6.222 (−0.444)
MMP2	_	_	_	_	−1.235 (−0.103)	−3.227 (−0.231)
MMP9	−5.624 (−0.352)	−5.236 (−0.327)	−4.694 (−0.293)	−3.901 (−0.244)	−5.243 (−0.437)	−4.793 (−0.342)
SRC	−6.497 (−0.406)	−5.730 (−0.358)	−2.170 (−0.136)	−5.927 (−0.370)	−5.251 (−0.438)	−6.038 (−0.431)

**Table 4:** Free energy (MM/GBSA) analysis for the interaction of ESR1 with Elemol.

System	∆G or ∆G_Bind_	∆G_Coulomb_	∆G_Covalent_	∆G_H-bond_	∆G_SA_ or ∆G_Sol_Lipo_	∆G_Solv_ or ∆G_SolGB_	∆G_Packing_	∆G_vdW_
ESR1–Elemol	−49.85	−31.73	2.89	−3.03	−8.09	30.21	−1.71	−38.39

## Data Availability

The data will be made available from the authors upon reasonable request.

## Supplementary Materials

The following supporting information can be downloaded at https://www.mdpi.com/article/10.3390/cimb46110705/s1, Figure S1: Network constructed for active compounds–potential targets; the pink nodes represent the selected phytocompounds of CT, the blue nodes represent the possible targets of compounds, and the edge represents the relation between two nodes. Figure S2: PPI network obtained from STRING database. Circles represent nodes, and edges represent the relation between nodes. Table S1: Ligand force field parameters. Table S2: Molecular interaction parameters for the binding of ligands to ESR1.
