# Supplementary material for: Integrative Network Pharmacology, Molecular Docking, and Dynamics Simulations Reveal the Mechanisms of Cinnamomum tamala in Diabetic Nephropathy Treatment: An In Silico Study
Source: Curr Issues Mol Biol. 2024 Oct 23;46(11):11868–89. doi: 10.3390/cimb46110705 (PMC11592827; doi:10.3390/cimb46110705)
Supplement: Supplementary file 1 [file cimb-46-00705-s001.zip › cimb-3189617-supplementary.pdf]

Article

Integrative Network Pharmacology, Molecular Docking, and Dynamics Simulation Reveal the Mechanisms of *Cinnamomum tamala* in Diabetic Nephropathy Treatment: An *In-silico* Study

Rashmi Singh [1,2], Nilanchala Sahu [3], Rama Tyagi [4], Perwez Alam [5,*], Ali Akhtar [5], Ramanpreet Walia [1], Amrish Chandra [3], and Swati Madan [1,*]

1. Amity Institute of Pharmacy, Amity University, Noida, Uttar Pradesh, 201303, India; srashmi8126@gmail.com (R.S.); rwalia@amity.edu (R.W); smadan3@amity.edu (S.M.)
2. Metro College of Health Sciences & Research, Greater Noida, Uttar Pradesh, 201310, India; srashmi8126@gmail.com (R.S.)
3. Sharda School of Pharmacy, Sharda University, Greater Noida, Uttar Pradesh, 201310, India; nilanchalasahu24@gmail.com (N.S.); amrish.chandra@sharda.ac.in (A.C.)
4. Galgotias College of Pharmacy, Greater Noida, Uttar Pradesh, 201310, India; tyagirama8@gmail.com (R.T.)
5. Department of Pharmacognosy, College of Pharmacy, King Saud University, P.O Box 2457, Riyadh 11451, Saudi Arabia. aperwez@ksu.edu.sa (P.A.); aakhtar@ksu.edu.sa (A.A.).

* Correspondence: smadan3@amity.edu (S.M.); aperwez@ksu.edu.sa (P.A.)

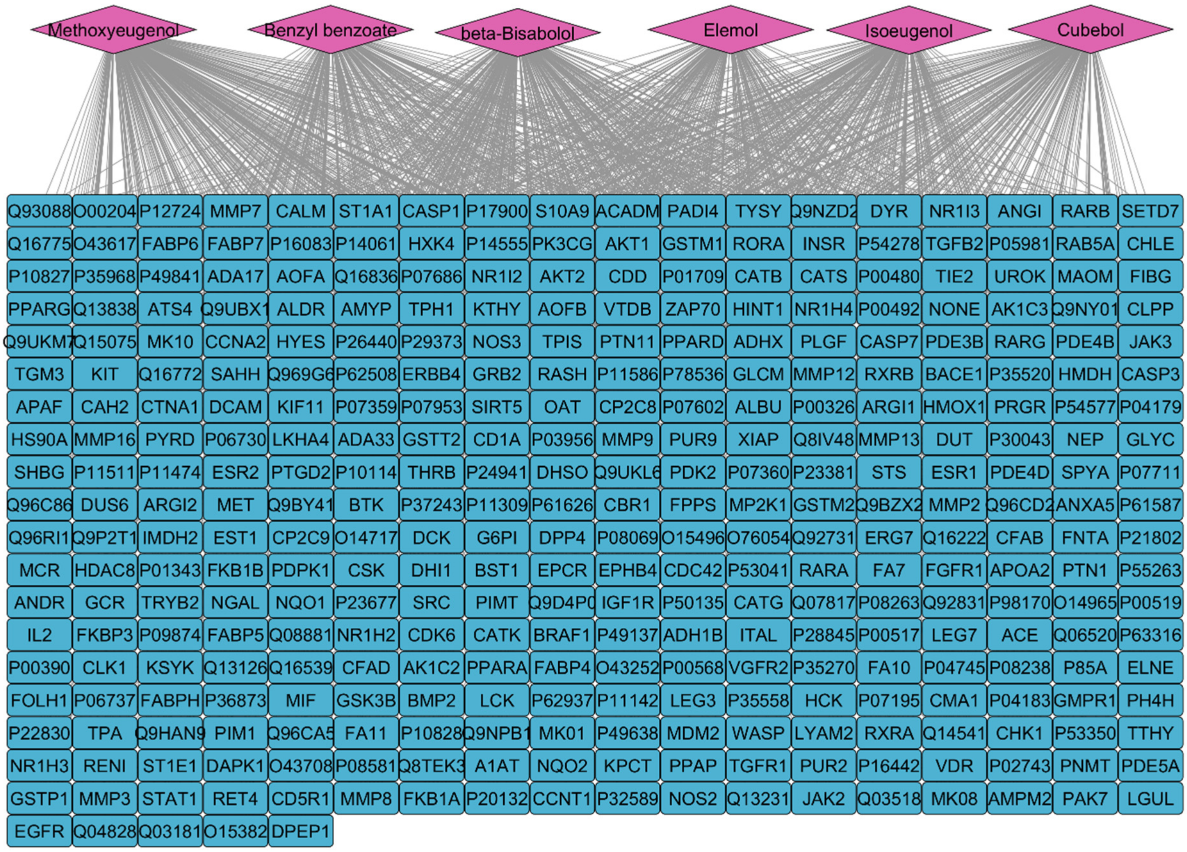

Figure S1. Network constructed for Active Compounds-Potential Targets, the pink nodes represent the selected phytocompounds of CT, the blue nodes represent the possible targets of compounds and the edge represents the relation between two nodes.

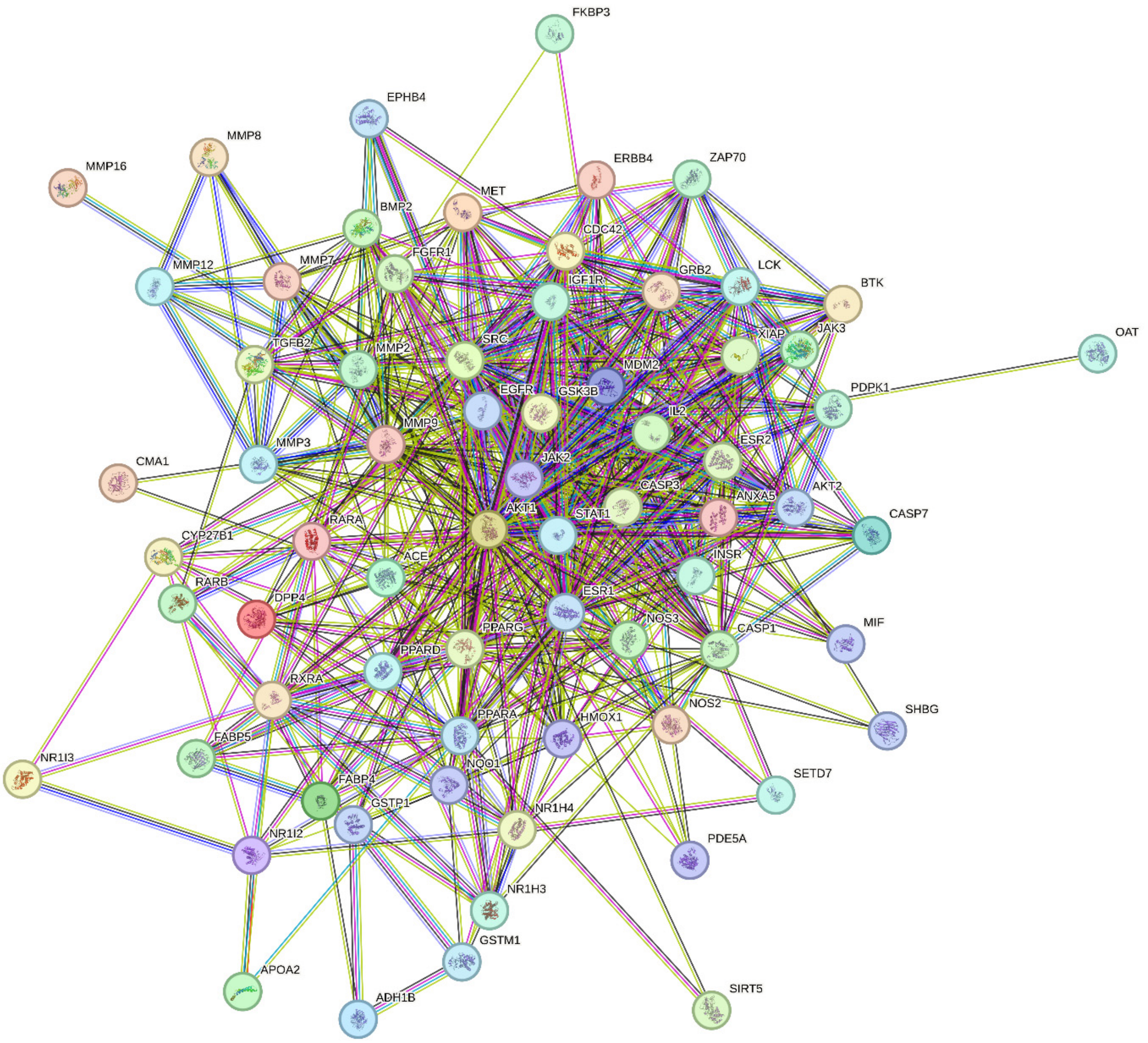

Figure S2. PPI network obtained from STRING database. Circles represent nodes and edges represent the relation between nodes.

Ligand Force Field Parameters:

The ligand force field parameters, including bond lengths, bond angles, dihedral angles, partial charges, and Lennard-Jones parameters, were generated using the CHARMM General Force Field (CGenFF). The partial charges were assigned using the RESP fitting method. The detailed parameters for the ligand are provided in Table S1.

Table S1: Ligand Force Field Parameters

Parameter Type	Atoms Involved	Value	Force Constant
Bond Length	C–N	1.47 Å	340 kcal/mol·Å^2
Bond Angle	N–C–C	120°	70 kcal/mol·rad^2
Dihedral Angle	C–N–C–O	180°	2 kcal/mol
Partial Charge	C	+0.25 e	—
Lennard-Jones	Atom C	σ = 3.5 Å	ε = 0.12 kJ/mol

Table S2. Molecular interaction parameters for the binding of ligands to ESR1.

Ligand	Donor-Acceptor pair	Distance (Å)	Type of Interaction
Control (Clomifene)	LIG:C - ASP351:OD1	3.3666	Carbon Hydrogen Bond
	LIG:C - ASP351:OD1	3.2638	Carbon Hydrogen Bond
	PHE404 - LIG	4.9608	Hydrophobic (Pi-Pi T-shaped)
	HIS524 - LIG	5.5409	Hydrophobic (Pi-Pi T-shaped)
	ALA350 - LIG:C	4.0402	Hydrophobic (Alkyl)
	LIG:C - LEU354	3.8124	Hydrophobic (Alkyl)
	LIG:C - LEU387	5.4482	Hydrophobic (Alkyl)
	LIG:C - LEU354	5.4882	Hydrophobic (Alkyl)
	LIG:C - PRO535	3.7678	Hydrophobic (Alkyl)
	TRP383 - LIG:C	4.2818	Hydrophobic (Pi-Alkyl)
	LIG - ALA350	3.7068	Hydrophobic (Pi-Alkyl)
	LIG - LEU525	5.0626	Hydrophobic (Pi-Alkyl)
	LIG - LEU346	5.4594	Hydrophobic (Pi-Alkyl)
	LIG - ALA350	4.8528	Hydrophobic (Pi-Alkyl)
	LIG - LEU387	4.8754	Hydrophobic (Pi-Alkyl)
	LIG - LEU391	5.0867	Hydrophobic (Pi-Alkyl)
	LIG - ILE424	5.1206	Hydrophobic (Pi-Alkyl)
	LIG - LEU525	5.1942	Hydrophobic (Pi-Alkyl)
Elemol	LIG:H - GLU353:OE1	2.9474	Conventional Hydrogen Bond
	LEU346 - LIG	4.6811	Hydrophobic (Alkyl)
	ALA350 - LIG	4.4396	Hydrophobic (Alkyl)
	ALA350 - LIG:C	4.4578	Hydrophobic (Alkyl)
	ALA350 - LIG:C	3.7887	Hydrophobic (Alkyl)
	LIG:C - LEU384	4.6969	Hydrophobic (Alkyl)
	LIG:C - MET343	5.3395	Hydrophobic (Alkyl)
	LIG:C - LEU525	4.7481	Hydrophobic (Alkyl)
	LIG:C - LEU384	5.3130	Hydrophobic (Alkyl)
	LIG:C - LEU525	4.5803	Hydrophobic (Alkyl)
	LIG:C - MET343	5.0055	Hydrophobic (Alkyl)
	LIG:C - LEU346	4.7359	Hydrophobic (Alkyl)
	LIG:C - MET421	4.6955	Hydrophobic (Alkyl)
	TRP383 - LIG:C	4.8061	Hydrophobic (Pi-Alkyl)
